# Halophiles and Their Vast Potential in Biofuel Production

**DOI:** 10.3389/fmicb.2019.01895

**Published:** 2019-08-22

**Authors:** Mohammad Ali Amoozegar, Atefeh Safarpour, Kambiz Akbari Noghabi, Tala Bakhtiary, Antonio Ventosa

**Affiliations:** ^1^Extremophiles Laboratory, Department of Microbiology, School of Biology and Center of Excellence in Phylogeny of Living Organisms, College of Science, University of Tehran, Tehran, Iran; ^2^Department of Industrial and Environmental Biotechnology, National Institute of Genetic Engineering and Biotechnology, Tehran, Iran; ^3^Department of Microbiology and Parasitology, Faculty of Pharmacy, University of Seville, Seville, Spain

**Keywords:** biofuel, halophile, biodiesel, biogas, bioethanol

## Abstract

Global warming and the limitations of using fossil fuels are a main concern of all societies, and thus, the development of alternative fuel sources is crucial to improving the current global energy situation. Biofuels are known as the best alternatives of unrenewable fuels and justify increasing extensive research to develop new and less expensive methods for their production. The most frequent biofuels are bioethanol, biobutanol, biodiesel, and biogas. The production of these biofuels is the result of microbial activity on organic substrates like sugars, starch, oil crops, non-food biomasses, and agricultural and animal wastes. Several industrial production processes are carried out in the presence of high concentrations of NaCl and therefore, researchers have focused on halophiles for biofuel production. In this review, we focus on the role of halophilic microorganisms and their current utilization in the production of all types of biofuels. Also, the outstanding potential of them and their hydrolytic enzymes in the hydrolysis of different kind of biomasses and the production of biofuels are discussed.

## Introduction

During the past years, the decrease in the availability of fossil fuels and environmental troubles have become major global problems, which has increased the focus and demand for alternative, environmentally friendly, and renewable energy resources (Kardooni et al., 2018). Biofuels are considered one of the best substitutes for fossil fuels and consequently, there is a growing interest in converting biomass to biofuel (Pimentel et al., 2009).

Some biofuels are produced directly from available food resources, like sugar, starch, and oil or from available crops, like sugar cane, corn, beets, wheat, sorghum, rapeseed, sunflower, soybean, palm, coconut, and Jatropha, which are recognized as first-generation biofuels, while second-generation biofuels are those which have been produced from raw materials with a difficult hydrolysis process, like lignocellulosic materials (Hirani et al., 2018). Utilization of the second-generation biofuels from non-food biomasses is more attractive because, during the process, biofuel production would not compete with food production while it reduces some of the environmental issues such as land and water and also economic costs such as energy consumption. However, the first-generation biofuels still do have their own importance (Begemann et al., 2011).

Biofuels are available in five types, including bioethanol, biobutanol, biogas, hydrogen, and biodiesel. Biodiesel and bioethanol are the main biofuels that are produced at industrial scales and >90% of total biofuel market is dedicated to them (Oh et al., 2018). Their success in this market is determined by various prerequisites defined by both chemical and physical properties. There are several chemical and thermo-chemical processes available for biofuel production but the biological conversion of biomass to biofuel by microorganisms is more cost-effective and has received great and extensive attention over the last years (Barnard et al., 2010). During the biofuel synthesis process, harsh conditions like the increase in pH and salt concentration occur which make the environment at conditions similar to alkaline and saline environments. Thus, microorganisms thriving under these conditions can possibly be used for biomass breakdown and biofuel production (Woolard and Irvine, 1995).

Halophiles can be found in hypersaline environments which are widely distributed in various geographical areas on Earth, such as saline lakes, salt pans, salt marshes, or saline soils. These microorganisms can be found in all three domains of life including Archaea, Bacteria, and Eukarya, and are distinguished by their requirement of high salinity conditions for growth. They may be classified according to the amount of their salt (NaCl) requirement: slight, moderate, and extreme halophiles which grow optimally at 0.2–0.85 M (1–5%), 0.85–3.4 M (5–20%), and 3.4–5.1 M (20–30%) of NaCl, respectively. In contrast, non halophilic microorganism grow optimally at <0.2 M (1%) NaCl concentrations. Halotolerant microorganisms are those that can grow in the presence and absence of high concentrations of salt (Singh et al., 2019). Halophilic and halotolerant microorganisms can grow over a broad range of salt concentrations, while requirement or tolerance for salts sometimes depends on environmental and nutritional factors; hence, making them the best choice for industrial processes especially for biofuel production (Margesin and Schinner, 2001). Furthermore, enzymes produced by halophilic microorganisms have an optimal function under very high salt concentrations, like KCl concentrations of ∼4 M or NaCl concentrations higher than 5 M. These enzymes have some extra amino acids which provide an extensive negative charge on the surfaces of the enzymes (Danson and Hough, 1997). Thus, the enzymatic effect is enhanced during biofuel production. In this review, we discuss the different types of biofuels and the role of halophilic microorganisms in their production. Moreover, we also discuss the potential of halophiles in biofuel production.

## Bioethanol/Biobutanol

Among all biofuels, bioethanol is frequently recognized as the most promising substitute and/or additive to gasoline which is why scientists have paid more attention to it (Sukumaran et al., 2005). Production of bioethanol via different enzymes from different biomasses is more eco-friendly and more popular than other processes. Lignocellulosic biomass or other plant biomasses are renewable materials, comprising mainly cellulose, hemicelluloses, lignin, and starch. Production of bioethanol from biomass consists of four major steps, including biomass pretreatment, enzymatic hydrolysis, fermentation, and distillation (Indira et al., 2018).

As cellulose, hemicelluloses and lignin are found in the rigid parts of plants and are highly resistant to biodegradation. To carry out fermentation reactions, it is necessary to pretreat this biomass at a high temperature or under extreme pH conditions (Mosier et al., 2005). Alkali pretreatment, for example with lime, has been used to treat wheat straw, poplar wood, switch-grass, and corn stover. Lime can be substituted by alkaline salts during pretreatment. The resulting pH and salt concentration makes the surrounding environment similar to alkaline–saline lakes. Hydrolyzed biomass generates reduced sugars, which are then converted to ethanol by microbial action. Because of this, halophiles and their enzymes could play critical roles in the mentioned stages (Woolard and Irvine, 1995). There are some reports about sugar fermentation and direct production of ethanol and butanol by halophiles. For example, Amiri et al. (2016) reported that the moderately halophilic bacterium, *Nesterenkonia* sp. strain F, isolated from Aran-Bidgol hypersaline lake in Iran, has the ability to produce butanol, and ethanol as well as acetone under aerobic and anaerobic conditions. This was the first report of butanol and ethanol production by a wild microorganism which does not belong to the class *Clostridia*. Also *Nesterenkonia* sp. strain F was the first halophilic strain shown to produce butanol under aerobic cultivation. Cultivation of *Nesterenkonia* sp. strain F under anaerobic conditions with 50 g/L of glucose for 72 h resulted in the production of 105 mg/L of butanol. Under aerobic conditions, through fermentation with 50 g/L initial glucose concentration, 66 mg/L of butanol and 291 mg/L of ethanol were produced. As shown in this study, the natural formation of butanol, which has been considered as exclusive to the *Clostridia* class, was also observed in *Nesterenkonia* sp. strain F, a halophile bacterium of the family *Micrococcaceae* of the order *Actinomycetales* under both, aerobic and anaerobic conditions (Amiri et al., 2016). In another study, a marine yeast, which was identified as *Candida* sp. was isolated, characterized, and utilized for bioethanol production using *Kappaphycus alvarezii*, red algal biomass. In this report, first, the ability and efficiency of the isolated marine yeast to grow and ferment sugar to ethanol in the presence of 2.5–15% salt concentration was validated by fermenting galactose in the presence of different salts at various concentrations. They showed that the yeast produced 1.23% of the ethanol from undiluted hydrolysate, with 5.5% of reduced sugar content and 11.25% of salt concentration after 72 h of incubation, which represents 50% of conversion efficiency. However, in the case of 3:1 diluted hydrolysate having 3.77% reduced sugar and 9.0% of salt content, 1.76% ethanol was obtained after 48 h, with 100% conversion efficiency. Similarly, in the case of 2:1 as well as 1:1 dilution, 100% conversion was observed within 48 h. Although this yeast had grown in the presence of 13% salt, its fermentation efficiency was relatively low at 11.25% salt concentration, which revealed the inhibitory effect of high salt content on fermentation. However, in the presence of 6.25–11.25% salt, conversion of sugar to ethanol was 100% (Khambhaty et al., 2013).

There are also shreds of evidence that show halophilic microorganisms could be used as non-food feedstocks for bioethanol production. For example, Aikawa et al. (2013) reported the direct conversion of the halophilic filamentous cyanobacterium *Arthrospira platensis* to ethanol without pretreatment or enzymatic hydrolysis processes. They indicated that *A. platensis* is a remarkable carbohydrate feedstock in the form of glycogen, which is a promising material for the production of bioethanol and other various commercially valuable chemicals. Prior to their study, ethanol was successfully produced at high yields (1.08 g/L per day) from non-pretreated cyanobacterial cells without adding any amylases, using an amylase-expressing strain of *Saccharomyces cerevisiae* and lysozyme. The total ethanol yield based on glycogen consumption was 86%, which is the highest yield of bioethanol from an oxygenic photosynthetic microorganism (Aikawa et al., 2013).

In addition, there are other feedstocks in which halophilic microorganisms could play roles in their hydrolysis process where the resulting products could be fermented to produce biofuels, especially bioethanol. Therefore, in the following sections, we described some of the important and frequent biomasses that could be used for bioethanol production.

### Starch

Starch is the reserved form of carbohydrate in plants and therefore is one of the most frequent biomasses on earth as it is highly produced by plants annually. In industrial applications, starch is hydrolyzed to produce glucose, glucose syrups, and high-fructose corn syrups. The resulting glucose could be fermented further to bioethanol (Bai et al., 2008). Among all the industries which proceed by using enzymes, the starch industry consumes 15–20% of the total amount, and α-amylase, β-amylase, glucoamylase, and glucose isomerase are the main enzymes in this industry (Bertoldo and Antranikian, 2002).

#### α-Amylase

α-Amylase (also known as endo-1, 4-α-D-glucan glucanohydrolase EC 3.2.1.1) cleaves α-1,4 linkages between adjacent glucose units of starch and produces glucose, maltose, and maltotriose to form linear amylose chains (Sivaramakrishnan et al., 2006). As mentioned above, the produced glucose could be a substrate for bioethanol production. The presence of this extracellular endoenzyme has been reported in several halophile microorganisms which are summarized in Table 1. This enzyme has been found in various groups of halophilic microorganisms, including mainly bacteria (Onishi and Hidaka, 1978; Onishi and Sonoda, 1979; Coronado et al., 2000; Deutch, 2002; Mijts and Patel, 2002; Amoozegar et al., 2003; Aygan et al., 2008; Kiran and Chandra, 2008; Prakash B. et al., 2009; Shafiei et al., 2010, 2011, 2012; Yamaguchi et al., 2011; Kumar and Khare, 2012; Li and Yu, 2012a, c; Uzyol et al., 2012), archaea (Kobayashi et al., 1992; Pérez-Pomares et al., 2003; Fukushima et al., 2005; Hutcheon et al., 2005; Moshfegh et al., 2013), fungi (Ali et al., 2014, 2015), marine bacteria (Qin et al., 2014), and Actinobacteria (Chakraborty et al., 2009, 2011). The molecular weight of these α-amylase enzymes varies from 30 to 140 kDa. Most of them can work properly in the presence of high concentrations of salt while some of them are active in a broad range of temperature and pH values (Table 1). Among them, the α-amylase from *Nesterenkonia* sp. strain F was interesting. Three of the amylase enzymes produced by this strain have been purified with molecular masses of 57, 100 and 110 kDa. These enzymes had a maximum activity at pH 6.5–7.5 and 40°C and in a broad range of NaCl concentrations (0–4 M), with optimal activity at 0.25 M NaCl. One of these amylases had the ability to metabolize starch, which makes it very important in Biotechnology. The activity of the enzymes was not hindered by Ca^2+^, Rb^+^, Li^+^, Cs^+^, Mg^2+^, and Hg^2+^, whereas Fe^3+^, Cu^2+^, Zn^2+^, and Al^3+^ strongly inhibited the enzyme activity. This α-amylase was inhibited by EDTA, though PMFS and β-mercaptoethanol had no inhibitory effect. This enzyme was also the first and only reported to increase the microbial α-amylase activity in the presence of organic solvents (Shafiei et al., 2010, 2011, 2012). Furthermore, as mentioned above, *Nesterenkonia* sp. strain F also has the ability to produce ethanol and butanol directly from glucose (Amiri et al., 2016). This suggests that with some modifications ethanol and butanol could be produced from starch only by utilizing this strain.

**TABLE 1:** Amylase enzymes (α-amylase, β-amylase and glucoamylase) from halophilic microorganisms^∗^.

	**Name of**			**Enzyme**				**Cloning of**
**Amylase type**	**microorganism**	**Type**	**Strain**	**size (kDa)**	**Enzyme stability and activity**			**enzyme**
					
					**Temperature range**	**pH range**	**NaCl range**	
					**(optimum) (°C)**	**(optimum)**	**(optimum) (M)**	
α-Amylase	*Haloferax mediterranei*^1^	H A		58	50–60 (60)	7–8	2–4 (3)	NR
	*Haloarcula* sp.^2^	H A	S-1	70	50	7	4.3	NR
	*Haloarcula hispanica*^3^	H A		43.3	37–60 (50)	3–9 (6.5)	0–5 (4–5)	NR
	*Halorubrum xinjiangense*^4^	H A		60	(70)	7–9 (8.5)	(4)	NR
	*Natronococcus* sp.^5^	H A	Ah-36	74	+50 (55)	6–8 (8.5)	2.5	+
	*Chromohalobacter* sp.^6^	H B	TVSP 101	72 and 62	30–85 (65)	(9)	0–3.4	NR
	*Salimicrobium halophilum*^7^	H B	LY20	81	30–80 (70)	6–12 (10)	0.4–3.4 (1.7)	NR
	*Thalassobacillus* sp.^8^	H B	LY18	31	30–90 (70)	6–12 (9)	0–3.4 (1.7)	NR
	*Halothermothrix orenii*^9^	M H B		52 and 72.3	(65)	(7.5)	To 4.5 (0.9)	+
	*Kocuria varians*^10^	M H B		77	NR	NR	1–2	+
	*Halomonas meridiana*^11^	M H B		NR	(37)	7–10 (7)	Up to 5.4 (1.7)	+
	*Marinobacter* sp.^12^	M H B	EMB8	72	(45)	6–11 (7)	0.1–3.4 (0.1)	NR
	*Acinetobacter* sp.^13^	M H B		55 and 65	(50–55)	(7)	(0.2–0.6)	NR
	*Micrococcus halobius*^14^	M H B		89	(50–55)	(6–7)	(0.25)	NR
	*Halomonas* sp.^15^	M H B	AAD21	NR	35–60 (50)	4–7 (6.5)	0.4	NR
	*Bacillus dipsosauri*^16^	M H B	DD1	30	(Below than 60)	(6.5)	NR	NR
	*Halobacillus* sp.^17^	M H B	MA-2	NR	50 (10–70)	7.5–8.5 (7.8)	(0.9)	NR
	*Bacillus* sp.^18^	M H B	TSCVKK	NR	40–70 (55)	6.5–9.5 (7.5)	1.7–3.4 (1.7)	NR
	*Bacillus* sp.^19^	M H B	AB68	NR	20–90 (50)	5–10.5 (10.5)	0–3.4 (0.9)	NR
	*Nesterenkonia* sp.^20^	M H B	F	100–106	(45)	6–7.5 (7.5)	0–4 (0.5)	NR
	*Zunongwangia profunda*^21^	M B		66	0–35 (35)	(7)	(1.5)	+
	*Saccharopolyspora* sp.^22^	M A	*A9*	66	(55)	8–12 (11)	1.8–2.6 (1.8)	NR
	*Streptomyces* sp.^23^	M A	D1	66	37–85 (45)	7–12 (9)	1.2–2 (1.2)	NR
	*Aspergillus penicillioides*^24^	H F	TISTR 3639	42	Below than 90 (80)	9–11 (9)	1.7–6.8 (5.1)	NR
	*Engyodontium album*^25^	H F	TISTR 3645	50	40–60 (60)	5–9 (9)	0–5.1 (5.1)	NR
β-Amylase	*Halobacillus* sp.^26^	M H B	LY9	NR	50–70 (60)	4–12 (8)	0.9–3.4 (1.7–2.1)	NR
	*Salimicrobium halophilum*^7^	M H B	LY20	81	(70)	6–12 (10)	0.4–3.4 (1.7)	NR
Glucoamylase	*Halolactibacillus* sp.^27^	H B	SK71	78.5	0–100 (70)	7–12 (8)	0–3.4 (1.3)	NR
	*Halorubrum* sp.^28^	H A	Ha25	140	(50)	(7–7.5)	0–4.5	NR
	*Alkalilimnicola* sp.^29^	H B	NM-DCM-1	80	47–57 (55)	8.5–10.5 (9.5)	1.7–2.6 (2)	+

*^∗^H A, halophilic archaeon; H B, halophilic bacterium; M H B, moderate halophile bacterium; M B, marine bacterium; M A, marine actinomycete; H F, halophilic fungi; N R, not reported. ^1^Pérez-Pomares et al. (2003), ^2^Fukushima et al. (2005), ^3^Hutcheon et al. (2005), ^4^Moshfegh et al. (2013), ^5^Kobayashi et al. (1992), ^6^Prakash B. et al. (2009), ^7^Li and Yu (2012a), ^8^Li and Yu (2012c), ^9^Mijts and Patel (2002), ^10^Yamaguchi et al. (2011), ^11^Coronado et al. (2000), ^12^Kumar and Khare (2012), ^13^Onishi and Hidaka (1978), ^14^Onishi and Sonoda (1979), ^15^Uzyol et al. (2012), ^16^Deutch (2002), ^17^Amoozegar et al. (2003), ^18^Kiran and Chandra (2008), ^19^Aygan et al. (2008), ^20^Shafiei et al. (2010, 2011, 2012), ^21^Qin et al. (2014), ^22^Chakraborty et al. (2011), ^23^Chakraborty et al. (2009), ^24^Ali et al. (2015), ^25^Ali et al. (2014), ^26^Li and Yu (2011), ^27^Yu and Li (2014), ^28^Siroosi et al. (2014), ^29^Mesbah and Wiegel (2018).*

#### β-Amylase

β-Amylase is an exoenzyme that hydrolyzes starch by removing maltose from the no reducing end of the starch. Unlike α-amylase, β-amylases are very rare. This enzyme is secreted by several species of the genus *Bacillus*, including *B. polymyxa*, *B. cereus*, and *B. megaterium*, and by *Clostridium thermosulfurogenes* (Kawazu et al., 1987). Up to now, the presence of β-amylase has been reported only in two halophile bacteria (Table 1). These two β-amylases are from two moderately halophilic bacteria, *Halobacillus* sp. strain LY9 (Li and Yu, 2011) and *Salimicrobium halophilum* strain LY20 (Li and Yu, 2012a). These enzymes showed activity under high temperatures and pH values and the optimal activity of both enzymes was at 1.7 M NaCl which revealed the potential of these β-amylases in industrial processes.

#### Glucoamylase

Glucoamylase (EC 3.2.1.3) is another starch hydrolyzing enzyme which catalyzes the sequential cleavage of α-(1,4) and α-(1,6) glycosidic bonds from the not reduced ends of starch and related oligosaccharides and produces glucose as the sole end product (Xu et al., 2016). Glucoamylases for industrial purposes are mainly produced from filamentous fungi, such as members of the genera *Aspergillus* and *Rhizopus*. However, industrial applications of fungal glucoamylases are often hampered by certain limitations such as moderate thermostability, acidic pH requirement, and slow catalytic activity that increase the process costs (Niehaus et al., 1999). Amylopullulanase is a type of glucoamylase (Ganghofner et al., 1998) with the ability to degrade α-1,4 glycosidic linkages in starch, amylopectin, and related oligosaccharides. A halophilic archaeon, *Halorubrum* sp. strain Ha25 was reported to produce an extracellular, halophilic, and organic solvent tolerant amylopullulanase (Siroosi et al., 2014). The molecular mass of the purified form of this enzyme was estimated to be about 140 kDa by SDS–PAGE. Both starch and pullulan were used as the substrate for this enzyme with *K*_m_ of 4 and 1.8 mg/mL, respectively. The optimum temperature for amylolytic and pullulytic activities was 50°C and the enzyme showed activity on 0–4 M NaCl (Table 1). The maximal amylolytic and pullulytic activity was at pH 7 and 7.5, respectively. This enzyme is very important in industry as it showed higher stability in the presence of non-polar organic solvents. Amylopullulanase has also been identified in the genome of the halophilic, alkalithermophilic isolate *Alkalilimnicola* sp. NM-DCM-1. PulD7 (the pullulanase) showed maximal activity at 55°C, pH 9.5, and 2 M NaCl and had good thermal stability (Table 1). This enzyme was resistant to organic solvents and hydrolyzed both starch and pullulan (Mesbah and Wiegel, 2018). Another report of a glucoamylase produced by a halophile is the glucoamylase from *Halolactibacillus* sp. strain SK71. This enzyme (Table 1) showed broad substrate specificity along raw starch digesting activity with excellent thermostable, alkali-stable, halo stable, and organic solvent-tolerant properties. Raw cornstarch was used for saccharification by the glucoamylase and subsequent ethanol production by *S. cerevisiae*. The yield of ethanol was 0.365 g/g of glucose consumed with 71.6% of the theoretical yield from raw starch (Yu and Li, 2014).

### Cellulose

Among all the available biomasses on Earth, cellulose is the most frequent one since it is a major component of plant tissues. Cellulose consists of D-glucose molecules (between several hundred to over ten thousand) connected by β-1,4 linkages (Zverlov et al., 2015). The availability of cellulosic biomass is estimated near 30 Gt per year via global terrestrial production which has resulted in the development of a very diverse, multi-faceted approach of cellulose biodegradation in the world (Badger, 2002). The cellulose degradation is usually accomplished by an inducible system of cellulolytic enzymes and proteins, working synergistically and comprising a distinct cellulase system (Wood, 1985). The cellulase complex (i.e., exoglucanase; EC 3.2.1.91, endoglucanase; EC 3.2.1.4, and β-D-glucosidase; EC 3.2.1.21) from cellulolytic microorganisms have been suggested as an acceptable strategy in the biotechnological conversion of cellulose. The cellulase works by hydrolyzing the β-1,4-glucosidic bonds between the glucosyl residues in cellulose (Béguin and Aubert, 1994). Of course, there are several cellulase producers in the microorganism world with higher activities but in high salt concentration conditions, halophiles with cellulase activity will be more useful. There are several reports of halophilic microorganisms showing cellulolytic activity; the screening of such powerful cellulose-producing microorganisms and using them in biofuel production is curtly a major focus of bioenergy research. Cellulase and other cellulytic enzymes hydrolyze cellulosic materials to sugars and these sugars are subsequently fermented to produce bioethanol and other bio-based products.

#### Cellulases From Halophilic Microorganisms in Biofuel Production

There are several reports of biofuel production from cellulolytic products using halophilic microorganisms. In a study, the haloarchaeon *Haloarcula* sp. strain LLSG7 with cellulolytic activity was isolated from the saline soil of Yuncheng Salt Lake, China. Cellulase production by this strain was strongly influenced by the salinity of the culture medium, where the maximum amount was obtained in the presence of 25% NaCl. Substrate specificity tests showed that the crude cellulase was a multicomponent enzyme system, and zymogram analysis revealed that strain LLSG7 secreted five different endoglucanases. The optimal cellulase activity of *Haloarcula* sp. strain LLSG7 was at 50°C, pH 8.0, and 20% NaCl. In addition, this cellulase was highly active and stable in broad ranges of temperature (40–80°C), pH (7.0–11.0), and NaCl concentration (17.5–30%). It displayed remarkable stability in the presence of non-polar organic solvents with log *P*_ow_ ≥ 1.97. The crude cellulase secreted by *Haloarcula* sp. strain LLSG7 was further utilized to hydrolyze alkali-pretreated rice straw. Then, the enzymatic hydrolysate was used as the substrate for bioethanol fermentation by *S. cerevisiae*. The production of bioethanol increased along the fermentation period and maximal yield of 10.7 g/L was observed after 30 h. No increase in ethanol production was obtained after 30 h. However, the concentration of total reduced sugars decreased along the incubation time and remained at about 1.4 g/L by the end of incubation. As reported, the obtained ethanol yield was about 0.177 g/g of dry substrate, with a conversion efficiency of 41.9%; these results were much higher than those reported from other fungal cellulases, which later were used in bioethanol fermentation with *S. cerevisiae* (Li and Yu, 2013). In another study, a novel halotolerant cellulolytic bacterium, *Bacillus methylotrophicus* RYC01101, was isolated from ruminant feces in Thailand. This strain could grow at 10% (w/v) NaCl. The cellulase activity of the bacterium on CMCase activity and FPase activity was reported at 0.230 ± 0.004 and 0.080 ± 0.007 U/mL, respectively. As for ethanol production, a pretreated cassava stalk (source of carbon with high cellulose content) was saccharified by the extracellular cellulase of *B. methylotrophicus* RYC01101 under incubation conditions for 72 h, yielding a cassava stalk hydrolysate containing 0.41 ± 0.01 mg/mL of glucose. This hydrolysate was subsequently co-cultured with *S. cerevisiae* TISTR 5111. At the end of the fermentation process, 1.38 ± 0.40 g/L of bioethanol was produced. However, further optimizations seem to be necessary (Chantarasiri, 2014). Another study was based on a halophilic bacterium *Gracilibacillus* sp. strain SK1 showing cellulolytic activity, that strain was isolated from Yuncheng Salt Lake. The salinity of the culture medium had a great influence on cellulase production of this strain with maximal levels of cellulase in the presence of 10% NaCl. The substrate specificity tests indicated that the crude cellulase is a multi-component enzyme system with a combined activity of endoglucanase, exoglucanase, and β-glucosidase. As zymogram analysis indicated, six different endoglucanases were secreted by this strain. They reported that the crude enzyme was highly active and stable over broad ranges of temperature (40–70°C), pH (6.0–10.0), and NaCl concentration (7.5–17.5%), with an optimum at 60°C, pH of 8.0, and 12.5% NaCl. These observations showed that this enzyme is splendidly thermostable, alkali-stable and halostable. Moreover, the crude cellulases of the *Gracilibacillus* sp. strain SK1 displayed high stability in the presence of hydrophobic organic solvents. For bioethanol production, first, the corn stover and rice straw were selected as the feedstocks; because the isolated cellulase was more effective against them. Maximal production of reduced sugar was obtained as 27.1 g/L in 48 h and 20.4 g/L in 64 h from corn stover and rice straw, respectively. Total amounts of reduced sugars released from 1 g of dry feedstock were 0.678 g/g (dry substrate) for corn stover and 0.502 g/g (dry substrate) for rice straw. Interestingly, it was observed that the considerable saccharification yield using the crude cellulase from *Gracilibacillus* sp. strain SK1 was close to some commercial enzymes, such as Celluclast, and Novozyme 188. *S. cerevisiae* was used for ethanol production from enzymatic hydrolysates of corn stover. The maximal yield of ethanol (13.5 g/L) was observed after 56 h of fermentation. Meanwhile, at the end of fermentation, the content of reduced sugars reached 0.9 g/L. The obtained ethanol yield was about 0.186 g/g (dry substrate), and the conversion efficiency of reduced sugars to ethanol was about 52.8%. These gained values were much higher than those reported for other fungal cellulases, which were usually used for bio-ethanol production (Yu and Li, 2015). Several halophiles with cellulolytic activity have been reported. Furthermore, several cellulases have been isolated and purified from halophilic microorganisms. Although no bioethanol production from these cellulases has yet been reported, their existence bolds the potential of halophiles in bioethanol production (Table 2). Cellulolytic enzymes from *Salinivibrio* sp. NTU-05 (Wang et al., 2009), *Bacillus* sp. BG-CS10 (Zhang et al., 2012), *Thalassobacillus* sp. LY18 3 (Li et al., 2012), *Bacillus* sp. L1 4 (Li and Yu, 2012c), and *Alkalilimnicola* sp. NM-DCM1 (Mesbah and Wiegel, 2017) have been purified. These enzymes showed interesting biochemical properties and have activity in the presence of organic solvents, high temperature, alkaline pH, and NaCl (Table 2).

**TABLE 2:** Purified cellulase enzymes from halophile bacteria^∗^.

	**Enzyme**				
**Name of microorganism**	**size (kDa)**	**Properties of the purified cellulase**	**Enzyme stability and activity**		
			**Temperature range**	**pH range**	**NaCl range**
			**(optimum) (°C)**	**(Optimum)**	**(Optimum) (M)**
*Salinivibrio* sp. NTU-05^1^	29	Halostable, Slightly thermostable	10–40 (35)	6.5–8.5 (7.5)	0–5 (0.9)
*Bacillus* sp. BG-CS10^2^	62	Thermostable, Salt tolerant, pH-tolerant	(55 and 35)^∗^	4.5–9.2 (5)	0–2.5 (2.5)
*Thalassobacillus* sp. LY18^3^	61	Organic solvent-tolerant, Alkali-stable	30–80 (60)	7–11 (8)	0.9–3 (1.7)
*Bacillus* sp. L1^4^	45	Organic solvent-tolerant, Thermostable, Alkali-stable, Halotolerant	30–80 (60)	7–9 (8)	0.5–2.6 (1.3)
*Alkalilimnicola* sp. NM-DCM1^5^	41	Halophilic, Alkali-thermostable, Ionic- liquid tolerant	45–60 (55)	7.5–10.5 (8.8)	1.2–2.9 (2.5)

*^∗^With and without NaCl. ^1^Wang et al. (2009), ^2^Zhang et al. (2012), ^3^Li et al. (2012), ^4^Li and Yu (2012b), ^5^Mesbah and Wiegel (2017).*

### Hemicellulose

Hemicelluloses are polysaccharides with heterogeneous and linear chains, and consist of 20–40% of lignocellulose (Marriott et al., 2016). Generally, in a lignocellulose complex, hemicellulose is covalently bound to lignin sheaths and interacts with cellulose through hydrogen bonds (Bailey et al., 1992; Joseleau et al., 1992). The composition of hemicellulose in a backbone structure, branching, and modifications differs greatly between plant species. Therefore, hemicelluloses are categorized based on the carbohydrate polymer composition including xylan (D-xylose), xyloglucan (D-xylose and D-glucose), glucomannan (D-glucose and D-mannose), galactoglucomannan (D-galactose, D-glucose and D-mannose) and arabinogalactan (D-galactose and L-arabinose). As hemicelluloses are polysaccharides with high molecular masses and rigid structures, it is necessary that multiple hemicellulases convert those large polymers into smaller oligosaccharides, disaccharides, and monosaccharides. In reality, the conversion of hemicelluloses requires esterases to remove acetyl and ferulic acid modifying groups and glycoside hydrolases to break the sugar backbone and branched sugar residues. Therefore, microorganisms involved in the degradation of hemicellulose are required to produce multiple enzymes with distinct specificity and function (Shallom and Shoham, 2003). Enzymes that hydrolyze hemicelluloses are diverse among mesophilic bacteria and fungi and interestingly, there are some reports involving extremophilic bacteria (Gilbert and Hazlewood, 1993; Shallom and Shoham, 2003; Badhan et al., 2007). In recent years, some novel halophilic archaea and bacteria with hemicelluloses degrading activity have been described and some halophilic and halotolerant hemicellulases have been purified. All of them are reviewed in the sections “Xylan” and “Mannan.”

#### Xylan

Among all hemicelluloses, xylan is the most frequent one in terrestrial plants (Jensen et al., 2018). As a heterogeneous polysaccharide, the backbone of xylan is composed of D-xylose linked by β-1,4 glycosidic bonds. Acetyl, arabinosyl, and glucuronosyl groups are often linked to the xylopyranoside units. Due to this fact, the xylan composition has large spectra among plant species. Two types of enzymes are required for xylan degradation, such as endo-β-xylanases (EC 3.2.1.8) which fragment the chain, and β-xylosidases (EC 3.2.1.37) that convert xylooligomers to monomers (Kulkarni et al., 1999). Besides, auxiliary enzymes like α-glucuronidase (EC 3.2.1.139), acetyl xylan esterase (EC 3.1.1.72), and ferulic and *p*-coumaric acid esterases (EC 3.1.1.73) are needed to remove side group residues (Begemann et al., 2011). Recently, several species of halophilic and halotolerant microorganisms with the ability to degrade xylan have been described, but still, there are no reports of them being used for biofuel synthesis.

Several xylanases and xylosidase enzymes have been characterized, isolated, and purified from halophilic microorganisms. They have been isolated from different types of microorganisms including: bacteria of the genera *Gracilibacillus*, *Chromohalobacter*, *Bacillus*, *Halomonas*, *Flammeovirga*, and *Marinimicrobium* and also *Halorhabdus* from the *Archaea* domain (Wainø and Ingvorsen, 2003; Wejse et al., 2003; Prakash S. et al., 2009; Giridhar and Chandra, 2010; Møller et al., 2010; Wang C. Y. et al., 2010; Yang et al., 2010; Cai et al., 2018). Xylanases from halophilic bacteria have a range of molecular masses from 15 kDa in *Chromohalobacter* sp. TPSV101 to 62 kDa in, strain CL8 (a halophile bacterium belonging to the Gammaproteobacteria) (Wejse et al., 2003; Prakash S. et al., 2009). In *Halorhabdus utahensis*, two xylanases were identified with molecular masses of 45 and 67 kDa (Wainø and Ingvorsen, 2003). All these xylanases showed activity in a broad range of physiological conditions such as pH, temperature, and also NaCl concentration. For example, the xylanase produced by the moderately halophilic bacterium *Gracilibacillus* sp. TSCPVG shows activity in the range from 0 to 30% of NaCl (Giridhar and Chandra, 2010). Furthermore, several xylosidases were either characterized from halophiles or their presence was inferred from the hydrolysate composition. Xylosidase is responsible for the complete degradation of xylan to D-xylose and it was identified in *Gracilibacillus* sp. TSCPVG and *H. utahensis* (Wainø and Ingvorsen, 2003; Giridhar and Chandra, 2010). β-1,3-Xylanase (Xyl512) was identified in the genome of the deep-sea bacterium *Flammeovirga pacifica* strain WPAGA1. This enzyme has optimal activity at 30°C, pH 7.5, and 1.5 M of NaCl. Furthermore, Xyl512 had activity at 20°C and pH 7.0 in the condition of no NaCl (Cai et al., 2018).

#### Mannan

Mannan is a homopolymer polysaccharide which is found greatly in cell walls of several species of algae and some plant seeds. D-Mannose monomers with β-1,4 glycosidic bonds have formed this hemicellulose (Singh et al., 2018). In soft trees, glucomannan, which is a heteropolymer of D-mannose and D-glucose, is more frequent (Whitney et al., 1998). Like xylanases, mannanases first produce oligomers from polymers and then by consequent hydrolysis, the monomeric sugars are formed (Dhawan and Kaur, 2007). The mannan and glucomannan hydrolysis enzymes are usually extracellular enzymes produced by some species in bacteria which include β-1,4 mannanase (EC 3.2.1.78), β-1,4 mannosidase (EC 3.2.1.25), and β-glucosidase (EC 3.2.1.21) (Dhawan and Kaur, 2007). There are some reports about halophiles with the ability to degrade mannan (Wainø and Ingvorsen, 1999; Møller et al., 2010; Wang J. et al., 2010). The first halo stable enzyme with mannan degradation ability was reported in 1999 from a novel and an extremely halotolerant *Bacillus* sp. strain NN, isolated from the Great Salt Lake, UT, United States (Wainø and Ingvorsen, 1999). They showed that this strain had the ability to produce β-mannanase and β-mannosidase in culture media containing at least 10% NaCl. They found that the β-mannanase and β-mannosidase had optimum activity at 1 and 5% of NaCl, respectively. However, the enzymes showed significant halo stability after incubation for 24 h at 20% NaCl and >50 and 100% of residual activity had been retained for β-mannanase and β-mannosidase, respectively. Furthermore, the β-mannanase activity of strain NN endured temperature and incubation at 60°C for 24 h in 10% NaCl had no effect on its activity. The other halo stable β-mannanase was isolated from the bacterium *Pantoea agglomerans* A021 and cloning and expression of this novel mannanase gene (man26P) have been carried out (Wang J. et al., 2010). The molecular mass of this enzyme was reported 38.5 kDa. Maximum activity of purified man26P was 514 U/mg, occurred at pH 6.0, and at a temperature of 55°C. This enzyme tolerated temperatures below 60°C and was stable upon exposure to buffers ranging from pH 4.0 to 10.0. The optimal activity in NaCl solutions makes it a very strong and suitable choice for industrial applications. Another report of mannan metabolizing activity was from the moderately halophilic bacterium, *Marinimicrobium haloxylanilyticum* strain SX15, isolated from the Great Salt Lake, with the ability to degrade some polysaccharides such as xylan, starch, carboxymethyl cellulose, and galactomannan (Møller et al., 2010). No further information was given in this report concerning the mannan degradation ability of this strain. As transient populations or normal microbiota, fungi are present in evaporator ponds and salterns where some of them have the ability to degrade plant biomass present in these hypersaline environments. *Scopulariopsis candida* strains LMK004 and LMK008 isolated from a solar saltern had the ability to use locust bean gum galactomannan as the carbon source in the presence of NaCl which shows β-mannanase production. The β-mannanase enzymes were partially purified and the molecular mass of LMK004 and LMK008 β-mannanases was 41 and 28 kDa, respectively. The maximum activity of LMK004 β-mannanase was at pH 5 and 50°C and 80% of it was retained at pH 5–6.5 after 24 h of incubation at 4°C. In contrast, only 60% of the β-mannanase activity of the LMK008 strain was retained at pH 6–7. Both enzymes lost their activity at temperatures below 40°C and only remained stable for 3 h between 30 to 40°C. High concentrations of NaCl were tolerated by LMK008 β-mannanase and after 2 h of incubation in 20% NaCl it showed 70% of activity whereas the β-mannanase of strain LMK004 was only active in NaCl concentrations between 0 and 10% (Mudau and Setati, 2008).

### Lignin

Lignin is the major structural component of cell walls structure in many plant species and makes it the second most abundant raw material on Earth. The presence of lignin provides strength and rigidity in plants and helps in water transportation (Kim et al., 2017). Heterogeneous polymers of lignin are made up from phenylpropanoid inter-units with covalent bonds and several isomers of lignin with complex structure exist. These rigid structures are resistant to degradation by most microorganisms (Li et al., 2009). Peroxidases and phenol oxidases are the major enzymes in lignin degradation under aerobic conditions. Phenoloxidases are further divided into two groups, including laccases and polyphenol oxidases (Datta et al., 2017). In anaerobic conditions, phenyl phosphate synthases and phenyl phosphate carboxylases are the main enzymes involved in lignin degradation. Several plants containing lignin live in coastal regions and salt marshes. Although our knowledge about lignin recycling in these environments is really limited, it seems that halophilic and halotolerant microorganisms play an important role in metabolizing lignin (Benner et al., 1986). On the other hand, lignin and lignin-based derivatives are the most abundant components in paper and pulp industries, tanneries, textile mills, and molasses-based distilleries. Because of poor biodegradation and intense color of lignin, it is considered as serious contamination in these industries (Raghukumar et al., 2008). It is notable that the waste streams in many of these industries usually contain high amounts of salts and thus it makes the halophilic and halotolerant microorganisms a good choice for lignin degradation. It is important to know that in the pulp and paper industries, water with high concentration of salts, especially NaCl, is recycled and halophilic and halotolerant enzymes could play important roles in biopulping (converting wood chips into pulp) and biobleaching (decolorizing by using enzymes) processes (Li et al., 2002). All these reasons support the importance of halophilic and halotolerant microorganisms in lignin degradation and reveal the possibilities of biofuel production from lignin. Furthermore, halophile microorganisms with phenol removal activities could decrease chemical oxygen demand (COD) parameters in industrial effluents according to the environmental standards (Moussavi et al., 2010). In this section, we report some halophilic bacteria and fungi showing the ability of lignin degradation.

#### Halophilic Microorganisms and Lignin Degradation

Prokaryotes are the most frequent organisms in the environments like anaerobic sediments, waterlogged wood, coastal seawater, and sediments and salt marshes. Therefore, in these environments, they play effective roles in the degradation of polymers like lignin (González et al., 1997a). *Sagittula stellata*, a marine aerobic bacterium, was the first halophilic strain with the ability to breaking down lignin into smaller units (González et al., 1997b). Among all the enzymes involved in lignin degradation, laccase is the most interesting one in environmental applications. This enzyme does not require additional components such as manganese or hydrogen peroxide for its activity. Also, in hypersaline conditions, it simultaneously breaks down lignin and decolorizes (Molitoris et al., 2000). In 2010, Uthandi et al. (2010) purified a laccase (LccA) from a halophilic archaeon, *Haloferax volcanii*. This enzyme tolerated high concentrations of salt from 0.1 to 1.4 M and high temperatures (55°C). It also had the ability to oxidize a wide range of organic substrates such as bilirubin, syringaldazine, etc. (Uthandi et al., 2010). Another laccase enzyme was purified from *Chromohalobacter* sp. and the molecular mass of this enzyme was estimated at 60 kDa. The optimal activity of this enzyme was at 3 M of NaCl, pH 8.0, and 45°C. The most effective inducer of laccase production of this strain was CuSO_4_ (Rezaei et al., 2014). A laccase enzyme was purified from a halotolerant endospore-forming bacterium, *Bacillus* sp. strain WT. This enzyme showed maximum activity at 100 mM NaCl and toward 2, 2′-azino-bis(3-ethylbenzothiazoline-6-sulfonate) (ABTS) and syringaldazine were at 55°C and pH values of 5.0 and 8.0, respectively. The potent laccase inhibitor, NaN_3_, had no effect on it in 1 mM concentration (Siroosi et al., 2016). The halophilic bacterium *Aquisalibacillus elongatus* had a highly stable extracellular laccase with the molecular mass of 75 kDa. This laccase was extremely stable against pH, temperature, and organic solvents (Rezaei et al., 2017). Laccase activity was also observed in a halophilic archaea and bacteria, *Bacillus safensis* sp. strain S31. Endospores of this bacterium were isolated from soil samples from a chromite mine in Iran. The maximum laccase activity was at 30°C and pH 5.0 using ABTS as the substrate (Siroosi et al., 2018). In Table 3, we have summarized the laccase enzymes from halophilic archaea and bacteria. A fungal laccase was reported from *Pestalotiopsis* sp. SN-3. This halotolerant fungus efficiently metabolized lignin and potentially degraded toxic substances from its surroundings. The laccase from *Pestalotiopsis* sp. SN-3 showed high activity and even tolerated high amounts of salt. *Pycnoporus sanguineus* produced two thermohalotolerant laccase isoforms with the ability to maintain their stability at high temperatures which increased their shelf-life (Dantán-González et al., 2008). Cloning of ligninolytic genes in halotolerant/halophilic microorganisms was another approach. For example, in this criterion genes encoding protocatechuate 3,4-dioxygenase were cloned in a marine *Bacillus* sp.; the isolated enzymes from this bacterium efficiently cleaved the bonds within the lignin (Żur et al., 2018). On the other hand, several halophilic fungi with powerful and extensive abilities to metabolize lignin have been reported. Some marine fungal species such as *Digitatispora marina*, *Halocyphina villosa*, and *Nia vibrissa* were found on decaying lignocellulosic substrates (Pang et al., 2011). In particular, it was shown that several enzymes with the ability to degrade lignin were produced by a marine halotolerant isolate, *Phlebia* sp. strain MG-60, which had been isolated from mangrove stands in Okinawa, Japan. The manganese peroxidase of this strain was expressed in different concentrations of NaCl and expression of it was regulated by the presence of Mn^2+^ (Kamei et al., 2008). Interestingly, the increase in Mn^2+^ or ${\text{NH}}_{4}^{+}$ inhibited the production of the enzyme but the addition of NaCl partially or completely reversed that inhibition (Li et al., 2003). Decolorization of the dye Poly R-478 is an indicator of laccase and manganese peroxidase activity and consequently lignin degradation, but in the case of *Phlebia* sp. strain MG-60, decolorization quality decreased when salt concentration increased. Furthermore, when the salt concentration increased, the strain MG-60 whitened unbleached pulps more efficiently than *Phanerochaete chrysosporium*, the well-studied white rot fungus with lignin degradation activity (Bucher et al., 2004). In addition, three fungus strains, *Ulomyces chlamydosporum*, *Emericella nidulans*, and *Aspergillus phoenicis*, isolated from the Dead Sea, showed very high potential as decolorizing agents. Moreover, some marine species such as *Ascocratera manglicola*, *Astrosphaeriella striatispora*, *Cryptovalsa halosarceicola*, *Linocarpon bipolaris*, and *Rhizophila marina* exhibited significant amounts of lignin solubilization (Molitoris et al., 2000).

**TABLE 3:** Purified laccase enzymes from halophilic archaea and bacteria^∗^.

	**Enzyme**							
**Name of microorganism**	**size (kDa)**	**ABTS oxidation**		**SGZ oxidation**		**Enzyme stability and activity**		
				
						**Optimum**	**Optimum pH**	**Optimum**
		***K*_m_ (μM)**	***k*_cat_ (s^–1^)**	***K*_m_ (μM)**	***k*_cat_ (s^–1^)**	**temperature (°C)**	**(for ABTS and SGZ)**	**NaCl (M)**
*Haloferax volcanii* strain SB01	75	700 and 671	10.0 and 9.9	67 and 35	19.4 and 21.7	45–50	6 and 8.4	1
and US02^1^								
*Chromohalobacter* sp.^2^	60	NR	NR	NR	NR	45	NR	3
*Bacillus* sp. strain WT^3^	180	132.7	309	3.7	51	55	5 and 8	0.1
*Aquisalibacillus elongatus*^4^	75	39.2	2150.0	16.1	918.8	40	6–8	2–3
*Bacillus safensis* sp. strain S31^5^	NR	NR	NR	NR	NR	30	5.5	0

*^∗^ABTS, 2,2′-Azino-bis (3-ethylbenzothiazoline-6-sulfonic acid); SGZ, syringaldazine; NR, not reported. ^1^Uthandi et al. (2010), ^2^Rezaei et al. (2014), ^3^Siroosi et al. (2016), ^4^Rezaei et al. (2017), ^5^Siroosi et al. (2018).*

## Biodiesel

During the past decades, biodiesel was regarded as an important alternative energy source because of the suitable properties and environmental benefits of it, and also because it is derived from biological resources (Azócar et al., 2010; Nejad and Zahedi, 2018). According to the catalysts engaged in the process, either chemical or enzymatic methods could produce biodiesel while the enzymatic process using lipases is more effective than the chemical methods (Antczak et al., 2009). Today, >95% of biodiesel is produced from edible oils, such as soybean oil, palm oil, and rapeseed oil, which may lead to the global imbalance of food supply and also may increase the cost of biodiesel production. Thus, biodiesel production from non-edible oils is a more logical approach (Yu et al., 2013). These non-edible oils include Jatropha oil and oils from halophilic microalgae (Abdullah et al., 2015). In the following sections, we have discussed the role of halophilic biomasses in biodiesel production; then, we review the role of halophilic microorganisms with lipase activity in biodiesel production.

### Halophiles as Biomass

#### Microalgae

Microalgae are known as the largest primary biomass that could be a safe and clean source of energy production in order to decrease global warming and environmental pollution (Tandon and Jin, 2017). Their high lipid content, in some cases up to 80% of their weight, high efficiency, fast growth, bio fixation of waste CO_2_, contribution to greenhouse preventing effects, and the possibility of being cultured on inappropriate farmlands, led to an increasing research interest on their use for biodiesel synthesis (Spolaore et al., 2006). *Dunaliella salina* is a halophilic green microalga found in saline environments such as saline lakes, salt ponds, and marine waters. *D. salina* produces high amounts of carotenoids which makes it a good source of food and antioxidant agents. Besides, it plays an important role in biodiesel production. Because of its high lipid content, especially linoleic and palmitic acids, *Dunaliella* is recognized as a good feedstock for biodiesel production. These fatty acids from *Dunaliella* would further get methylated to produce biodiesel (Rasoul-Amini et al., 2014). In a recent study, the ability of 21 halophilic microalgae, isolated from the hypersaline Bardawil lagoon, was evaluated in order to induce lipid production. Among all the isolates, a green microalga *Tetraselmis elliptica*, having the high lipid production capacity and predominant fatty acid contents of palmitic acid (C16:0) and oleic acid (C18:1n-9), was suggested as a potential source for biodiesel production (Abomohra et al., 2017).

On the other hand, halophilic microalgae *Dunaliella* sp. has been employed as a favorable feedstock for bioethanol production. The data indicated that the acidic pretreatment of the microalgal biomass of *Dunaliella* sp. using diluted sulfuric acid (1%) enhanced the bioethanol production level up to 7.26 g/L, which was 10.7 times higher than the level obtained from untreated biomass (Karatay et al., 2016). Several studies have shown the effect of different chemical factors on growth and lipid accumulation in microalgae (Yeesang and Cheirsilp, 2011). Among them, salinity is one of the most important factors and microalgae cells are directed toward energy storage, particularly lipid synthesis rather than an active growth under salt stress. Bartley et al. (2013) examined the growth and lipid synthesis of marine microalgae, *Nannochloropsis salina* along with deleterious algae opponents within an open culture system at different percentages of salinity. They observed that the highest algal growth and biomass occurred at the salinities of 22 and 34 PSU, while the minimum density of harmful opponent organisms was achieved at 22 PSU. In order to determine whether lipid synthesis reaches the maximum level under salinity stress, *N. salina* was cultivated at a concentration of 22 PSU, allowing the cells to reach the stationary growth phase and then increased the salinity to 34, 46, and 58 PSU. Interestingly, they found that lipids accumulate at higher salinities and with a maximum at 34 PSU (36% dry mass) (Bartley et al., 2013).

While it is still unclear how microalgae cells develop their selective response to salt stress on cellular and molecular levels, what matters the most now is to find a convenient and reliable approach for enhancing lipid production in marine microalgae after exposure to salt stress. Some simple examples of such adaptive responses of microalgae cells may include the changes in their morphology, physiology, and biochemical processes, as they occur only with salt-induced stress (Kirrolia et al., 2011). It has been revealed that hypersaline cyanobacterial species accumulate glycine betaine as the compatible solute for osmoregulation. In contrast, freshwater cyanobacteria such as *Synechococcus* species can develop an adaptive strategy following an increase in external salt concentration, arising the soluble sugars accumulation (Mackay et al., 1984; Reed et al., 1986). The osmolyte glycine betaine is highly consistent in cytoplasmic activities, enabling the cells to preserve membrane elasticity against denaturation by Na^+^ and other antagonistic ions in high osmolality conditions (Papageorgiou and Murata, 1995). Efforts have also been made to generate a genetically engineered freshwater cyanobacterium, *Synechococcus* sp. PCC 7942, with glycine betaine synthesis ability, allowing it to grow faster under high saline conditions compared to the untransformed cells (Nomura et al., 1995). As for another example, overexpression of BetT protein, known as the betaine transporter from a halotolerant alkaliphilic cyanobacterium *Aphanothece halophytica*, in *Synechococcus* cells resulted in NaCl-activated betaine uptake activities with enhanced salt tolerance (Laloknam et al., 2006). Previously, it has been shown that salt-stress tolerance in *A. halophytica*, as a halotolerant alkaliphilic cyanobacterium, could be related to the existence of an additional Na^+^-dependent F_1_F_0_-ATP_ase_ in the cytoplasmic membrane (Soontharapirakkul et al., 2011). Miriam et al. (2017) have recently reported that the halophilic microalgae *A. halophytica* can be a promising feedstock for bioenergy generation. This cyanobacterium with adaptive plasticity in response to various environmental conditions could grow maximally at 60 ppt salinity, 0.05 g L^–1^ (N), 0.5 g L^–1^ (P), and 0.5 g L^–1^ (K). The favorable and general properties of this strain, like the high capacity to produce lipids with a low free fatty acid content, are the most potent and promising reasons for it be considered as an alternative way of clean energy production (Miriam et al., 2017).

### Lipase From Halophiles

As a ubiquitous hydrolytic enzyme, lipases (EC 3.1.1.3) have several applications in biotechnological and industrial fields, especially in biodiesel production (Hama et al., 2018). These enzymes catalyze the reverse reactions in non-aqueous solvent systems and along with the oil–water interface, they hydrolyze triglycerides into glycerol and fatty acids (Teo et al., 2003). Bacteria and fungi are the main producers of lipases in the industry (Sharma et al., 2011). Lipases from halophilic microorganisms have their own valuable characteristics. These enzymes could work properly in the harsh conditions in most industrial processes. Thus, screening for novel lipases from halophiles may be a proper approach (Amoozegar et al., 2008). So far, only two lipases from halophiles have ever been used for biodiesel production. One of these lipases was purified from the halophilic bacterium *Idiomarina* sp. W33. The molecular mass of this organic solvent-tolerant extracellular lipase was about 67 kDa. A substrate specificity test indicated that this enzyme preferentially hydrolyzes the long-chained *p*-nitrophenyl esters. The lipase from strain W33 had optimal activity at 60°C, pH 7.0–9.0, and 10% NaCl and could remain stable over a broad range of temperatures (30–90°C), pH values (7.0–11.0), and NaCl concentrations (0–25%). This lipase was thermostable, alkali-stable, and halotolerant. Diethyl pyrocarbonate and phenylarsine oxide inhibited the enzyme activity. Therefore, histidine and cysteine residues are important in the active site of it. In the presence of hydrophobic organic solvents, this lipase exhibited high stability and activity with log *P*_ow_ ≥ 2.13. Lipase from strain W33 also was used for biodiesel production from Jatropha oil. The yield of the free and immobilized form of lipase was 84 and 91%, respectively (Li et al., 2014). Usually, free lipases have low biodiesel production because they get aggregated in low water media and have caused mass transfer problems (Shah and Gupta, 2007). Biodiesel synthesis from immobilized lipases might be due to their larger surface area (Noureddini et al., 2005). Another lipase from halophiles was purified from *Haloarcula* sp. G41. This haloarchaeal strain was isolated from the saline soil of Yuncheng Salt Lake, China. The molecular mass of the purified lipase was 45 kDa where the salinity of the medium strongly affected the production of lipase. Maximum production of lipase was achieved in the presence of 20% NaCl or 15% Na_2_SO_4_. It preferred long-chained *p*-nitrophenyl esters. The lipase from the strain G41 showed thermostable, alkali-stable, and halostable properties and also high activity and stability over a broad range of temperature (30–80°C), pH (6.0–11.0), and NaCl concentration (10–25%), with an optimum at 70°C, pH 8.0, and 15% NaCl. Like the lipase from *Idiomarina* sp. W33, this lipase is a metalloenzyme where serine and cysteine residues are essential for its function. In the presence of hydrophobic organic solvents, this enzyme showed high stability and activity with log *P*_ow_ ≥ 2.73. Application of this lipase was assayed in biodiesel production. Free and immobilized forms of the lipase from the strain G41 reached in yield 80.5 and 89.2%, respectively (Li and Yu, 2014). In addition to these lipases, several other lipase enzymes from halophilic microorganisms were isolated and characterized. Finally, several screenings have been carried out in order to isolate new halophiles with lipase activity (Amoozegar et al., 2008; Rohban et al., 2009; de Lourdes Moreno et al., 2016; Esakkiraj et al., 2016; Ameri et al., 2017; Gutiérrez-Arnillas et al., 2017; Ai et al., 2018).

## Biogas

### Hydrogen

Among different energy sources, hydrogen (H_2_) has attracted great attention. Because of its easy conversion to electricity and clean combust, biological ways of hydrogen production are the new interest of many scientists (Gao et al., 2018). The favorite biological producers of H_2_ are the photosynthetic microorganisms (green algae, cyanobacteria, and photosynthetic bacteria) and non-photosynthetic bacteria (nitrogen-fixing bacteria and anaerobic bacteria) (Balat and Balat, 2009). Several studies have reported the production of H_2_ by halophilic photosynthetic and non-photosynthetic bacteria. In the case of photosynthetic bacteria, Ike et al. (1999) showed that a community of halophilic bacteria originated from night soil treatment sludge, strongly produced H_2_ from raw starch in the light and in the presence of 3% NaCl. The effective H_2_ producing strains of the community were *Vibrio fluvialis*, *Rhodobium marinum*, and *Proteus vulgaris*. The levels of H_2_ produced from starch by co-culture of *V. fluvialis* and *R. marinum* were nearly equal to the bacterial community, indicating the major role of these two halophile bacteria on H_2_ production from starch. Further observations have demonstrated that in pure culture, *V. fluvialis* produced acetic acid and ethanol from the degradation of starch and it appeared like that the strain *R. marinum* used this material for H_2_ production in bacterial communities or co-cultures. However, the pure culture of *R. marinum* in a synthetic medium containing acetic acid and ethanol couldn’t produce H_2_, suggesting that *V. fluvialis* supplied both substrates and some unknown factors for H_2_ production by *R. marinum*. Furthermore, these co-cultures were used for H_2_ production from two microalgae, *Chlamydomonas reinhardtii* and *Dunaliella tertiolecta*. The results showed a high yield of H_2_ production from these starch-rich biomasses. The advantage of the photosynthetic bacteria in H_2_ production is their low cost as they are able to produce it in the presence of the light from non-food biomasses or agricultural wastes. This study has shown the role of halophiles in this low-cost process (Ike et al., 1999). In another study, a new species of the family *Vibrionaceae*, *Vibrio* sp. showed the highest hydrogen yield (with 90% efficacy) at the highest NaCl concentrations (7.5%) under dark conditions. This hydrogen production occurred in a microbial community under moderate salinity conditions, suggesting new possibilities of technological development for treating saline effluents and producing biohydrogen (Pierra et al., 2014). *Vibrio tritonius* strain AM2 is the other halophilic strain with the ability to produce hydrogen from glucose and mannitol and powdered brown macroalgae containing 31.1% dry weight of mannitol. This strain was isolated from the gut of a marine invertebrate and yielded 1.7 mol H_2_/mol mannitol at pH 6 and 37°C. Compared to glucose, mannitol might be a better substrate for bioH_2_ production using strain AM2, which showed its ability to produce hydrogen from non-food feedstocks. Fermentation product profiling showed that this strain might be utilizing the formate-hydrogen pathway for hydrogen production (Matsumura et al., 2014). In a study, the production of biohydrogen by soil bacteria was observed under high salt concentrations (26% NaCl). This finding is important as it indicates that H_2_ producing bacteria can be found in hypersaline environments. The requirements to Cl^–^ ions were also observed in these bacteria (Taroepratjeka et al., 2019).

#### Glycerol and Biohydrogen

In hypersaline environments, glycerol plays an important role. Some halophiles accumulate organic solutes like glycerol in their cytoplasm to overcome the pressure of high salt concentration in their surroundings (Oren, 2008). For example, the green algae of the genus *Dunaliella* are the main producers of glycerol in hypersaline environments worldwide. Some member of the order *Halanaerobiales* has the ability to metabolize glycerol and produce important products (Oren, 2017). In the biodiesel industry, glycerol is produced as a by-product which often contains inhibitory factors for microorganisms such as heavy metals and salts (Johnson and Taconi, 2007). The advantage of the halophilic bacteria, in this case, is that contamination like this is not a major problem for them, because they have been reported to be heavy metal resistant and high salt concentrations cause no problem for their growth (Nieto et al., 1989). On the other hand, growing halophiles in high salt concentrations could lower the sterilization costs in the glycerol production process as several non-halophilic microorganisms cannot live under such high salt conditions (Ventosa and Nieto, 1995).

Glycerol-based hydrogen production by halophilic bacteria has been reported from *Halanaerobium saccharolyticum* subspecies *saccharolyticum* and *senegalensis*. These anaerobic, Gram-negative strains were isolated from the sediments of hypersaline lakes and belong to the order *Halanaerobiales* (Cayol et al., 2002). Hydrogen, carbon dioxide, and acetate were the main metabolites of glycerol fermentation of both strains. The highest hydrogen yields were achieved with 2.5 g/L glycerol and 150 g/L salt at pH 7–7.4 (Kivistö et al., 2010). To improve the hydrogen yield of *H. saccharolyticum* subsp. *saccharolyticum*, the genome of this strain has been sequenced. Following the genome sequence analysis, the glycerol fermentation pathways of this bacterium were reconstructed. This reconstruction revealed that the putative fermentation products were hydrogen, carbon dioxide, acetate, butyrate, butanol, ethanol, lactate, malate, and 1,3-propanediol (a vitamin B_12_-dependent route) and four [FeFe]-hydrogenases, two of them putative bifurcating hydrogenases requiring both reduced ferredoxin and NADH, were identified. The putative bifurcating hydrogenases are suggested to be involved in the high-yielded H_2_ production. Furthermore, the genes for a multidrug efflux pump (Acr type), β-lactamase, mercuric reductase, a copper-translocating ATPase, and a cobalt–zinc–cadmium-resistant protein were identified which means that *H. saccharolyticum* should be resistant to a wide variety of antibiotics and toxic compounds, including heavy metals (Kivistö et al., 2013).

### Methane

Methane (CH_4_) biogas is assumed as a renewable fuel for generating heat and electricity. A diverse community of microbes (mainly bacteria and methanogens) could convert the biomasses like livestock manure, crop residues, food wastes, food-processing wastes, municipal sludge, and municipal solid wastes to methane via anaerobic digestion. In the absence of oxygen, microorganisms hydrolyze the polymers of biomasses and the resulting hydrolysis products further get fermented to short chain fatty acids (SCFA), H_2_, and CO_2_, then archaeal methanogens ultimately convert them to methane biogas (a mixture of CH_4_ and CO_2_) (Yu and Schanbacher, 2010). Marine macroalgae can be used as biomass for the production of biomethane (Wei et al., 2013). But the disadvantage is that marine macroalgae contain salts (Roesijadi et al., 2010) which leads to inhibition of microbial activity in macroalgae-based methane production (Oren, 1999). By diluting the salinity, some studies have reported the production of methane (Vergara-Fernández et al., 2008; Costa et al., 2012; Hinks et al., 2013; Jard et al., 2013). Albeit, it seems that the production of methane under non-diluted conditions is more advantageous. At high salt concentrations, the production of methane is stronger and lower amounts of water are needed (Schramm and Lehnberg, 1984). Using this method, Miura et al. (2014, 2015a,b, 2016) have reported a series of experiments in which they modified the production of methane from brown algae by the methanogenic microbial community from marine sediments under high salinity (Miura et al., 2014, 2015a,b, 2016). In one of their studies, the predominant bacterial and archaeal strains with methane production activity belonged to the family *Fusobacteriaceae* and the genus *Methanosaeta*, respectively (Miura et al., 2015b).

## Conclusion

**FIGURE 1:**
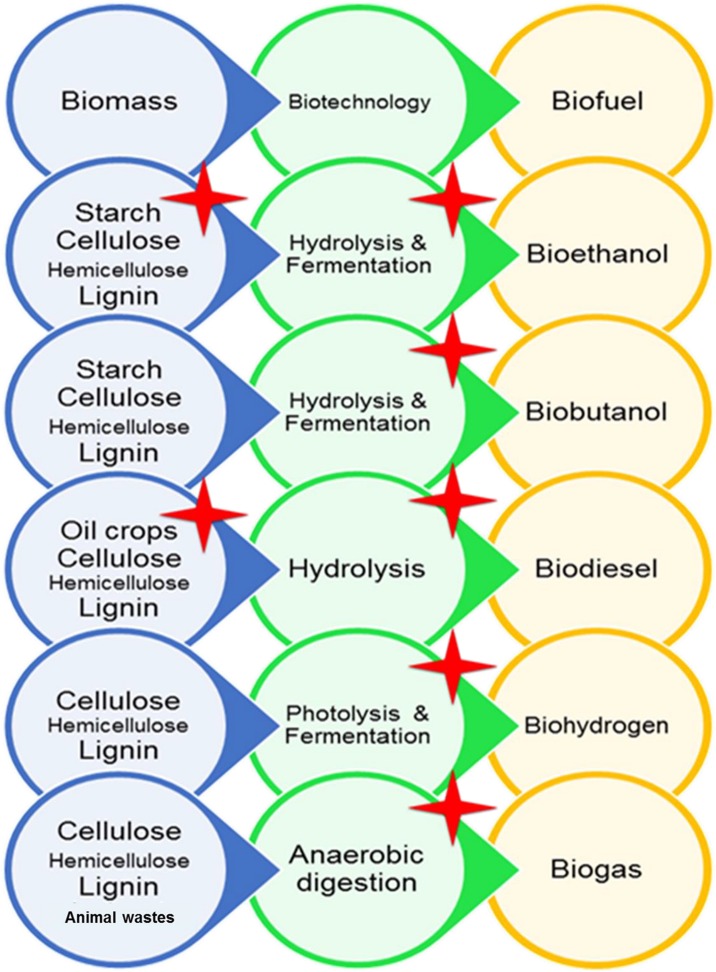
Different ways of production of different types of biofuels. Red stars show the processes in which the effect of halophilic microorganisms is reported in biofuel production.

Energy is a fundamental issue which determines the technological progress and the living standards of a society in the entire world. The fossil fuels play important roles in current international relations, but several analyses have shown that reserves of these old fuels are not sustainable. On the other hand, excessive utilization of these fuels in recent years have caused global warming and environmental pollutions. Therefore, an urgent need and research have been evoked to develop new fuels. Biofuels are the best choice because they usually are produced from low-cost materials in biological processes. Utilization of microorganisms in biofuel production has altered it into an easy and commercially viable process. As we had discussed earlier, there are several types of biofuels which are produced from several and different food and non-food feedstocks. Microorganisms could not only be a part of biofuel production by performing biotechnological processes, but also some types of microorganisms are considered as a feedstock for biofuel production. Generally, during industrial processes, harsh conditions such as high or low temperatures, pH, and salinity occur, and utilization of microorganisms with extraordinary abilities could improve biofuel production ways and lower the costs of these biotechnological applications. Thus, extremophiles and their enzymes are regarded as good choices for biofuel production. Halophiles are extremophiles with the ability to grow under high salt concentrations or amounts of NaCl. Halophiles and their enzymes have unique features, and this makes them attractive for both science and industry. Halophilic enzymes have the ability to catalyze several metabolic reactions, while other enzymes cannot. Several enzymes from halophiles could be active in the presence of high salt concentrations or other extreme conditions which makes them adequate for industrial processes. Up to now, a great number of halophilic microorganisms have been identified and several biotechnological applications have been reported from these microorganisms. Several metabolizing enzymes like amylases, lipases, cellulases, or chitinases have been purified from these microorganisms. Some of these enzymes are extremozymes and stable or active in a broad range of temperature, pH, and salts. This rich pool of extreme enzymes is so attractive for all the industries that they rely on for biotechnology and especially for biofuel production. The utilization of microorganisms is highly beneficial in biofuel production and utilization of halophiles may improve. In Figure 1, the pathways of biofuel production are demonstrated, and halophilic microorganisms have the great potential to be involved in any part of this map. Several halophiles potentially are suitable to be used as feedstocks to produce bioethanol, biobutanol, biohydrogen, and biodiesel. As summarized in Table 4, a few halophiles have been investigated and certainly, growing numbers are expected. Furthermore, several hydrolysis enzymes from halophilic microorganisms are being utilized in biomass degradation for the consequent production of bioethanol or biodiesel (Table 4). As we have demonstrated in this review, a great number of hydrolysis enzymes with extraordinary characteristics from halophiles exist that potentially could play important roles in biofuel production. In the fermentation stage of bioethanol and biobutanol production, halophiles are gradually finding suitable positions, while more research might be helpful to accelerate this process. In biofuel production from lignocellulosic materials, there are possibilities to get novel cellulases which degrade lignocellulosic biomass without pretreatment. Moreover, there is a possibility to find halophiles with both cellulase activity and phenol removal activity to decrease COD concentration and environmental pollution. Also, halophilic microorganisms with their special abilities have an undeniable role in the production of biohydrogen and biogas. Among all mentioned above, it seems that the halophilic microorganisms could create a brighter future for the production of biofuel and more investigations are needed to employ and support the vast potential of halophiles in biofuel production.

**TABLE 4:** Current halophilic microorganism used in biofuel production.

	**Halophilic microorganism**	**Hydrolysis of biomass or**	
**Type of biofuel**	**as biomass**	**biotechnological applications**	**Fermentation processes or biofuel production**
Bioethanol			*Nesterenkonia* sp. strain F^1^
			*Candida* sp.^2^
	*Arthrospira platensis*^3^		
		*Halolactibacillus* sp. SK71^4^	
		*Haloarcula* sp. strain LLSG7^5^	
		*Bacillus methylotrophicus* RYC01101^6^	
		*Gracilibacillus* sp. strain SK1^7^	
Biobutanol			*Nesterenkonia* sp. strain F^8^
Biodiesel	*Dunaliella salina*^9^		
	*Aphanothece halophytica*^10^		
		*Idiomarina* sp. W33^11^	
		*Haloarcula* sp. G41^12^	
Biohydrogen			*Rhodobium marinum* co-culture with *Vibrio fluvialis*^13^
			*Vibrio* spp.^14^
			*Vibrio tritonius* strain AM2^15^
			*Halanaerobium saccharolyticum*^16^
Methane biogas			Strain from the family *Fusobacteriaceae*^17^
			Strains from the genus *Methanosaeta*^17^

*^1^Amiri et al. (2016), ^2^Khambhaty et al. (2013), ^3^Aikawa et al. (2013), ^4^Yu and Li (2014), ^5^Li and Yu (2013), ^6^Chantarasiri (2014), ^7^Yu and Li (2015), ^8^Shafiei et al. (2010, 2011, 2012), ^9^Rasoul-Amini et al. (2014), ^10^Miriam et al. (2017), ^11^Li et al. (2014), ^12^Li and Yu (2014), ^13^Ike et al. (1999), ^14^Pierra et al. (2014), ^15^Matsumura et al. (2014), ^16^Cayol et al. (2002), ^17^Miura et al. (2015b).*

## Author Contributions

MA and AV conceived the review. AS and MA developed the theory and wrote the manuscript. AV revised the manuscript. KN revised the sections related to microalgae. TB and AV helped AS in language editing of the manuscript. MA supervised the work. All authors revised the final version of the manuscript.

## Conflict of Interest Statement

The authors declare that the research was conducted in the absence of any commercial or financial relationships that could be construed as a potential conflict of interest.
